# Endoscopic submucosal dissection for early esophageal squamous cell carcinoma: long-term results from a Western cohort

**DOI:** 10.1055/a-2245-7235

**Published:** 2024-02-07

**Authors:** Ilse N. Beaufort, Charlotte N. Frederiks, Anouk Overwater, Lodewijk A.A. Brosens, Arjun D. Koch, Roos E. Pouw, Jacques J.G.H.M. Bergman, Bas L.A.M. Weusten

**Affiliations:** 1 Department of Gastroenterology and Hepatology, University Medical Center Utrecht, Utrecht University, Utrecht, Netherlands; 2 Department of Gastroenterology and Hepatology, St. Antonius Hospital, Nieuwegein, Netherlands; 3 Department of Pathology, University Medical Center Utrecht, Utrecht University, Utrecht, Netherlands; 4 Department of Gastroenterology and Hepatology, Erasmus MC Cancer Institute, University Medical Center, Rotterdam, Netherlands; 5 Department of Gastroenterology and Hepatology, Amsterdam University Medical Centers, Amsterdam, Netherlands

## Abstract

**Background** Although endoscopic submucosal dissection (ESD) is established as first-choice treatment for early esophageal squamous cell carcinoma (ESCC) worldwide, most data are derived from Asian studies. We aimed to evaluate the long-term outcomes of ESD for patients with early ESCC in a Western cohort.

**Methods** In this retrospective cohort study, patients with early ESCC amenable to ESD were included from four tertiary referral hospitals in the Netherlands between 2012 and 2017. All ESD procedures were performed by experienced endoscopists, after which the decision for additional treatment was made on a per-patient basis. Outcomes were curative resection rate, ESCC-specific survival, and overall survival.

**Results** Of 68 included patients (mean age 69 years; 34 males), ESD was technically successful in 66 (97%; 95%CI 93%–100%), with curative resection achieved in 34/66 (52%; 95%CI 39%–64%). Among patients with noncurative resection, 15/32 (47%) underwent additional treatment, mainly esophagectomy (n = 10) or definitive chemoradiation therapy (n = 4). Endoscopic surveillance was preferred in 17/32 patients (53%), based on severe comorbidities or patient choice. Overall, 31/66 patients (47%) died during a median follow-up of 66 months; 8/31 (26%) were ESCC-related deaths. The 5-year overall and ESCC-specific survival probabilities were 62% (95%CI 52%–75%) and 86% (95%CI 77%–96%), respectively.

**Conclusion** In this Western cohort with long-term follow-up, the effectiveness and safety of ESD for early ESCC was confirmed, although the rate of noncurative resections was substantial. Irrespective of curative status, the long-term prognosis of these patients was limited mainly due to competing mortality.

## Abstract (de)

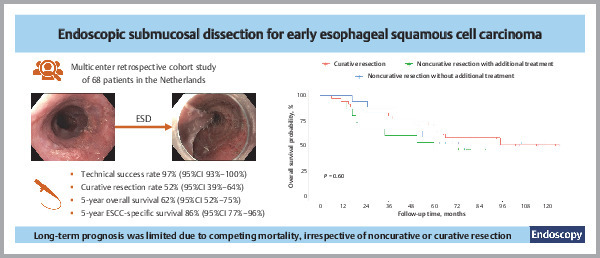

## Introduction

Esophageal cancer is the eighth most common cancer worldwide in terms of incidence 1 . The two main histopathological subtypes are esophageal squamous cell carcinoma (ESCC) and esophageal adenocarcinoma, which are characterized by a strikingly different geographic distribution. While ESCC is relatively uncommon in Europe and Northern America 2 , this subgroup is predominant in Eastern Asia and accounts for 85%–90% of all esophageal cancers globally 1 3 .

Current guidelines recommend endoscopic submucosal dissection (ESD) as first-choice treatment for superficially spreading ESCC to enable an en bloc resection with accurate histopathological staging while also potentially being curative 4 5 6 . For high grade intraepithelial neoplasia and T1m2 cancers (i.e. maximum invasion into the lamina propria), with good to moderate differentiation and absence of lymphovascular invasion, ESD is considered to be curative because the related risk of lymph node metastases is low, with a reported incidence of 0%–6% (i.e. “very low risk curative resection”) 6 7 8 9 10 11 . ESCC invading the muscularis mucosa (T1m3) or the superficial (i.e. ≤200 µm) submucosa (T1sm1) may also be curatively resected with ESD if the tumor is well to moderately differentiated and lymphovascular invasion is absent (i.e. “low risk curative resection”) 6 .

As a direct consequence of the low incidence of ESCC in Western countries, most data regarding outcomes of ESD for ESCC are derived from Asian series. This emphasizes the need for more data from Western populations, particularly with long-term follow-up, to guide further clinical decision making. Hence, our study aimed to evaluate the long-term outcomes of ESD for patients with early ESCC in a Western cohort.

## Methods

### Study population

This retrospective cohort study was conducted in four tertiary referral hospitals in the Netherlands. We included patients between January 2012 and December 2017 who had a lesion that was suspected of being ESCC and was considered amenable to ESD. Informed consent for ESD was obtained from all patients.

### ESD procedure

Prior to the ESD procedure, a separate endoscopic imaging procedure was generally performed by one of the participating expert endoscopists to assess whether ESD was considered technically feasible and potentially curative. During this imaging endoscopy, both high definition white-light and virtual chromoendoscopy (narrow-band imaging or blue-light imaging) were used to assess the extent of the lesion and to estimate the depth of invasion based on macroscopic features as well as microvascular patterns. In addition, Lugol’s iodine staining was used at the discretion of the treating endoscopist. No standardized tumor staging procedures to exclude advanced carcinoma (i.e. >T2) or lymph node metastasis were performed prior to the ESD procedure. However, the treating endoscopist could opt for endoscopic ultrasonography (EUS), computed tomography (CT) of the chest/abdomen, and/or positron emission tomography (PET)-CT, particularly when features suspicious for deep submucosal invasion were present.

**Fig. 1:**
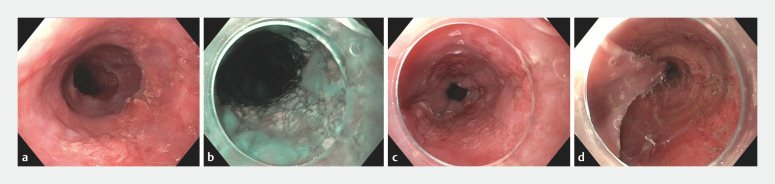
Endoscopic submucosal dissection (ESD) of an early esophageal squamous cell carcinoma (ESCC). **a** This ESCC lesion was classified as a type 0-IIa and 0-IIc involving 75% of the esophageal circumference. **b** The lesion was delineated using virtual chromoendoscopy. **c** Coagulation markings were then placed using the tip of the ESD knife. **d** The resection scar after ESD covered approximately 4 cm in length. Histopathological evaluation showed a radically removed, poorly differentiated, T1m3 tumor without lymphovascular invasion.

All ESD procedures were performed by dedicated endoscopists who were specifically trained in this technique and who carried out more than 20 ESD procedures per year. After detailed inspection of the lesion using high definition chromoendoscopy, the area of resection was delineated with electrocoagulation markings ( Fig. 1 ). Thereafter, a lifting agent selected by the performing endoscopist was injected to enable submucosal lifting. Mucosal incision and dissection were subsequently performed using DualKnife (Olympus, Tokyo, Japan), ITknife2 (Olympus), or HybridKnife (Erbe Elektromedizin GmbH, Tübingen, Germany), depending on the endoscopist’s preference.

Anticoagulants were discontinued prior to the procedure according to local guidelines, except for acetylsalicylic acid or carbasalate calcium.

High dose proton pump inhibitors (i.e. 40 mg twice daily) were prescribed to all patients who underwent an ESD in the distal part of the esophagus. In addition, patients with an ESD extending more than 75% of the esophageal circumference typically received either corticosteroid injections (i.e. triamcinolone) at the resection scar or oral corticosteroids (i.e. prednisolone or budesonide) to prevent esophageal stricture formation.

### Histopathological assessment

The ESD specimens were pinned on paraffin and fixed in formalin. The fixed specimens were then sectioned at 3–4 mm intervals and processed according to routine clinical care. All specimens were assessed by experienced gastrointestinal pathologists.

### Additional treatment and follow-up

The decision for additional treatment following ESD depended on the final histopathological diagnosis, in accordance with the European Society of Gastrointestinal Endoscopy (ESGE) guideline 6 . In general, endoscopic follow-up was recommended after an ESD with histology no more advanced than high grade intraepithelial neoplasia or T1m2. For radically resected T1m3/sm1 tumors, endoscopic follow-up was preferred if high risk features (i.e. poor tumor differentiation or lymphovascular invasion) were absent, while additional treatment was recommended in cases with high risk features. Additional treatment was also advised in cases of nonradical resection or deep submucosal invasion (i.e. ≥T1sm2). The final decision about additional treatment was made on a per-patient basis after discussion in a multidisciplinary meeting, taking into account the patient’s age, comorbidities, and preference.

For patients entering endoscopic follow-up, the first follow-up endoscopy was scheduled 3–6 months after ESD, and usually annually thereafter. Endoscopic follow-up entailed detailed inspection of the resection area as well as the entire esophagus with high definition virtual chromoendoscopy and histopathological sampling in cases of any visible abnormality. EUS, CT chest/abdomen, and/or PET-CT were additionally performed to detect metastatic recurrence if indicated on a per-patient basis.

### Outcomes and definitions

For this study, short-term outcomes after ESD were as follows: (1) technical success rate, defined as the percentage of patients in whom the ESD was technically complete; (2) en bloc resection rate, defined as the percentage of patients in whom the target lesion was resected in a single piece; (3) radical resection rate, defined as the percentage of patients in whom the target lesion was resected en bloc, with both vertical and lateral margins free of carcinoma; (4) curative resection rate, defined as the percentage of patients with a curative endoscopic resection; and (5) adverse event rate, defined as the percentage of patients with esophageal stricture, post-procedural bleeding, perforation, or any other adverse event leading to hospitalization or prolongation of existing hospitalization after ESD. A curative resection was defined in accordance with the ESGE guideline: (1) an en bloc radical resection of a superficial ESCC with histology no more advanced than intramucosal cancer (T1m2), well to moderately differentiated and without lymphovascular invasion (i.e. very low risk curative resection); or (2) an en bloc radical resection of an ESCC T1m3 or T1sm1 tumor that is well to moderately differentiated without lymphovascular invasion (i.e. low risk curative resection).

In addition, long-term outcomes were evaluated, which included: (1) incidence of metachronous lesions; (2) recurrence rate, defined as the percentage of patients with local recurrence (i.e. recurrent neoplasia within 1 cm of original endoscopic resection scar), locoregional and/or distant metastases during endoscopic follow-up after endoscopic resection; (3) ESCC-specific survival; and (4) overall survival.

### Data collection and data management

Patients were identified from a prospective database at each participating site. Dedicated research fellows collected all relevant data in a standardized database by reviewing the electronic patient files. All endoscopy and pathology reports and further clinical information were reviewed for data collection. Data concerning development and treatment of recurrence, as well as date and cause of death were checked with general practitioners and referring hospitals. All fields were examined for missing data, strange values, or outliers, with completion or correction where possible.

### Statistical analysis

For descriptive statistics, continuous variables were expressed as means with SD or medians with minimum and maximum values (min–max) or 25th and 75th percentiles (p25–p75) where appropriate. Categorical variables were presented as frequencies and percentages of the total. Outcome variables were reported with adjusted 95%CI obtained with simple bootstrapping with 1000 samples. For overall and disease-specific survival analyses, follow-up time started from the date of the ESD procedure until the date of death or end of follow-up (31 December 2022). For recurrence-free survival, the follow-up time between the ESD procedure until the date of recurrence or the last follow-up endoscopy was used. Overall and recurrence-free survival were estimated using the Kaplan–Meier method.

Data were analyzed with the statistical software packages IBM SPSS statistics version 26 for Windows (IBM Corp., Armonk, New York, USA) and R version 4.2.2 for Windows (R Foundation for Statistical Computing, Vienna, Austria), and the statistical significance level was set at *P* < 0.05.

### Ethics

The Medical Research Ethics Committee NedMec declared that this study was not subject to the Medical Research Involving Human Subjects Act. In addition, the need for formal patient informed consent was waived. Where possible, electronic patient files were checked for registration of objection to participation in research. The study was approved by the institutional review boards of the four participating hospitals.

## Results

### Patients

**Fig. 2:**
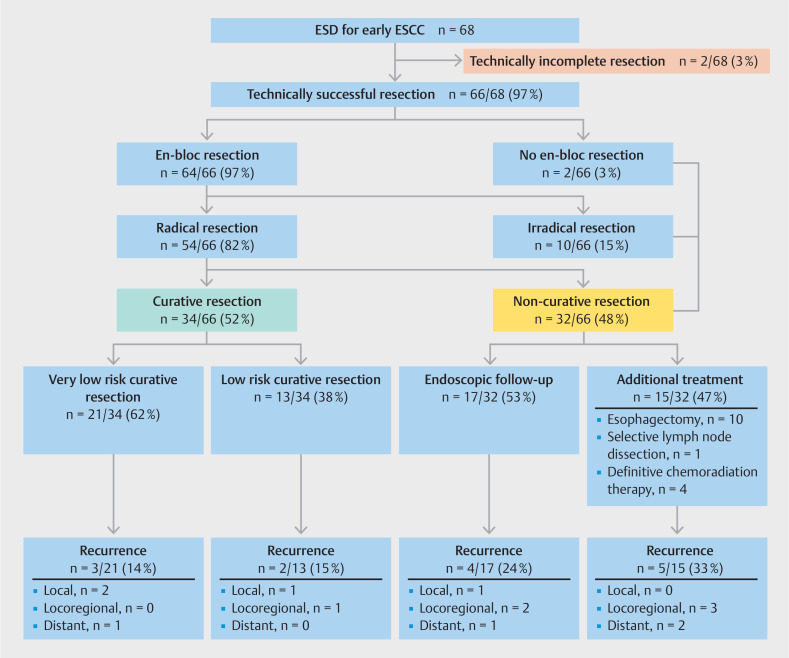
Flow chart of outcomes after endoscopic submucosal dissection (ESD) for early esophageal squamous cell carcinoma (ESCC). Radical resection was defined as an en bloc resection with tumor-free vertical and lateral margins. Very low risk curative resection was defined as a radical resection of high grade intraepithelial neoplasia or a well to moderately differentiated T1m2 tumor without lymphovascular invasion. Low risk curative resection was defined as radical resection with histopathology no more advanced than T1sm1, with good to moderate differentiation and no lymphovascular invasion.

The complex and heterogeneous outcomes of our cohort are easier to comprehend when the flow chart in Fig. 2 is used as the backbone of this Results section. A total of 68 patients were included after ESD ( Fig. 2 ), and baseline characteristics are summarized in Table 1 . In brief, half of the patients were male and mean age of patients was 69 years (SD 9). The main indications for diagnostic gastroscopy were complaints of dysphagia (18/68; 26%), retrosternal chest pain (17/68; 25%), and weight loss (9/68; 13%), while the remaining 15 patients (22%) underwent diagnostic gastroscopy for other indications.

**Table:** **Table 1** Baseline characteristics of included patients.

Baseline characteristics	Patients (n = 68)
**Patient characteristics**	
Age, mean (SD), years	69 (9)
Male, n (%)	34 (50)
ASA classification, n (%)	
I	10 (15)
II	40 (59)
III	18 (26)
IV	0 (0)
Anticoagulant use, n (%)	31 (46)
**Tumor characteristics**	
Primary Paris component, ^1^ n (%)	
0-Is	8 (12)
0-Ip	1 (2)
0-IIa	35 (51)
0-IIb	21 (31)
0-IIc	3 (4)
Secondary Paris component, ^2^ n (%)	
0-Is	2 (3)
0-Ip	2 (3)
0-IIa	9 (13)
0-IIb	17 (25)
0-IIc	13 (19)
Not applicable	25 (37)
Tumor length in cm, n (%)	
<3	20 (29)
3–5	38 (56)
6–8	6 (9)
>9	4 (6)
Circumferential extent in %, n (%)	
<25	5 (7)
25–49	21 (31)
50–74	17 (25)
75–99	12 (18)
100	13 (19)
Pre-ESD tumor staging, n (%)	
PET-CT	19 (28)
CT chest/abdomen	35 (51)
EUS	27 (40)
Ultrasound neck	22 (32)
ASA, American Society of Anesthesiologists; ESD, endoscopic submucosal dissection; PET-CT, positron emission tomography-computed tomography; CT, computed tomography; EUS, endoscopic ultrasonography. ^1^ Main type of morphology. ^2^ In lesions with combined morphology (e.g. 0-IIa + 0-IIc).	

Median tumor size was 3 cm (min–max 1–12) with a median circumferential extent of 50% (min–max 10–100). The majority of lesions were classified as 0-IIa lesions (51%) or 0-IIb lesions (31%) according to the Paris classification. Pre-resection tumor staging procedures were performed in 48 patients (71%), although staging procedures were very heterogeneous ( Table 1 ). In two patients (3%), lymph node metastases were suspected on (PET-)CT shortly after the ESD procedure. In addition, a few patients (4/68; 6%) underwent ESD despite suspicious endoscopic features of deep submucosal invasion (i.e. ≥T1sm2). This decision was made based on the fact that ESD was the only treatment option after these patients were considered ineligible for surgery owing to severe comorbidities.

### ESD outcomes

As shown in Fig. 2 , ESD was technically successful in 66/68 patients (97%; 95%CI 93%–100%). In one patient, the ESD procedure could not be completed due to perforation, for which urgent surgical esophagectomy was required. In another patient, ESD had to be aborted due to a severe laceration, which was managed by stent placement. These two patients with technically incomplete ESD were subsequently excluded from further analysis. En bloc resection was achieved in 64/66 technically complete ESDs (97%; 95%CI 93%–100%). The median total procedure time was 108 minutes (p25–p75 76–160) ( Table 2 ).

**Table:** **Table 2** Endoscopic submucosal dissection and histopathological characteristics of patients who underwent a technically successful endoscopic resection procedure.

	Patients (n = 66)
**ESD characteristics**	
Lugol’s iodine staining during imaging endoscopy, n (%)	36 (55)
Type of ESD knife, n (%)	
DualKnife ^1^	38 (58)
DualKnife and ITknife ^1^	2 (3)
HybridKnife ^2^	26 (39)
Procedure duration, median (p25–p75), minutes	108 (76–160)
Hospitalization, n (%)	57 (86)
Hospitalization duration, median (p25–p75), days	1 (1–2)
Complications, n (%)	
Post-procedural bleeding	1 (2)
Perforation	2 (3)
Infection	1 (2)
Stricture	21 (32)
**Pathology characteristics**	
Invasion depth, n (%)	
HGIN	15 (23)
T1m2	8 (12)
T1m3	15 (23)
T1sm1	8 (12)
T1sm2	10 (15)
T1sm3	10 (15)
Differentiation grade, n (%)	
Good	12 (18)
Moderate	28 (42)
Poor	10 (15)
No differentiation	1 (2)
Not applicable	15 (23)
Lymphovascular invasion, n (%)	
No	37 (56)
Yes	14 (21)
Not applicable	15 (23)
Tumor-negative vertical resection margins, n (%)	58 (88)
Tumor-negative lateral resection margins, n (%)	59 (89)
ESD, endoscopic submucosal dissection; HGIN, high grade intraepithelial neoplasia; p25–p75, 25th to 75th percentile; T1m2, invasion in lamina propria; T1m3, invasion in muscularis mucosae; T1sm1, submucosal invasion ≤200 µm; T1sm2, submucosal invasion ≤500 µm; T1sm3, submucosal invasion >500µm. ^1^ Olympus, Tokyo, Japan. ^2^ Erbe Elektromedizin GmbH, Tübingen, Germany.	

Adverse events occurred in 23/66 successful procedures (35%; 95%CI 19%–46%). Perforations occurred in two patients (2/66; 3%). In one patient, the perforation could be treated conservatively with antibiotic treatment, whereas stent placement was necessary to resolve the other perforation. Esophageal stricture was the most common adverse event, with an incidence of 32% (21/66; 95%CI 17%–49%). The majority of patients with a resection scar extending more than 75% of the esophageal circumference received either corticosteroid injections or oral corticosteroids (21/24; 88%). Despite these preventive measurements, 14 of the 21 patients (67%) with an esophageal stricture had a resection scar with a circumferential extent of more than 75%. Most strictures could be managed endoscopically, with a median number of 6 dilations (p25–p75 3–15). In addition, four patients (4/21; 19%) required stent placement and one patient (1/21; 5%) underwent incisional therapy.

### Histopathological outcomes

Histopathological assessment revealed 54/66 resections (82%; 95% CI 73-91%) with tumor-free resection margins, of which 53/66 were en-bloc resections resulting in a radical resection rate of 80% (95% CI 71-90%). Tumor-positive vertical and lateral resection margins were found in 8/66 (12%) and 7/66 (11%) patients, respectively. A curative resection was achieved in 34/66 patients (52%; 95%CI 39%–64%), of which 21/34 resections (62%) were very low risk and 13/34 (38%) were low risk ( Fig. 2 ). The distribution of tumor invasion depth is shown in **Table 2** .

### Additional treatment

None of the 34 patients with a curative resection underwent adjuvant therapy, whereas additional treatment was performed in 15/32 patients (47%) with a noncurative resection ( Fig. 2 ). One of these patients was diagnosed with a lymph node metastasis on PET-CT. Owing to severe comorbidities, this patient was additionally treated with a selective lymph node dissection instead of esophagectomy. The remaining patients underwent either an esophagectomy with or without neoadjuvant (chemo)radiation therapy (n = 10) or definitive (chemo)radiation therapy (n = 4). In this specific subgroup undergoing step-up treatment, the majority of endoscopic resection specimens (9/15; 60%) revealed multiple high risk features (see **Fig. 1s** in the online-only Supplementary material); a few cases had only one high risk feature of invasion depth ≥T1sm2 (n = 5) or presence of lymphovascular invasion (n = 1).

In the remaining patients with a noncurative resection (17/32; 53%), endoscopic surveillance was chosen over additional treatment strategies because of severe comorbidities or patient preference ( Fig. 2 ). As shown in **Fig. 1s** , the majority of these ESD specimens (11/17; 65%) contained only one high risk feature (nonradical resection n = 5, poor differentiation n = 2, lymphovascular invasion n = 2, or deep submucosal invasion n = 2).

Ablation therapy (i.e. radiofrequency ablation, cryoballoon ablation, or argon plasma coagulation) for synchronous or metachronous flat-type intraepithelial neoplasia was performed in five patients (5/66; 8%).

### Recurrence rate

All patients entered follow-up, with a median duration of 66 months (p25–p75 40–86). Patients underwent a median of 7 endoscopies (p25–p75 3–12) during follow-up, with the last endoscopy at a median of 40 months (p25–p75 14–60) after ESD.

Metachronous lesions were detected in eight patients (8/66; 12%; 95%CI 4%–20%) at a median of 15 months (p25–p75 8–35) after ESD. Three of these metachronous lesions (3/8; 38%) developed after a noncurative resection, while five metachronous lesions (5/8; 62%) were detected after a very low risk (n = 2) or low risk (n = 3) curative resection. The majority (7/8; 88%) could be treated endoscopically with either endoscopic mucosal resection (n = 4), ESD (n = 2), or cryoballoon ablation (n = 1). One patient developed a locally advanced metachronous lesion (T4aN0M0) 34 months after prior noncurative resection and was subsequently treated by palliative radiation therapy.

In total, four patients (4/66; 6%; 95%CI 0–12%) developed local recurrence at a median of 13 months (p25–p75 10–25) after ESD. One local recurrence (1/4; 25%) occurred after a noncurative resection, and three local recurrences (3/4; 75%) were detected after a curative resection (very low risk n = 2, low risk n = 1; Fig. 2 ). Overall, the local recurrence could be treated endoscopically in three patients (3/4; 75%), either with endoscopic mucosal resection (n = 2) or radiofrequency ablation (n = 1). The other patient had a local recurrence not amenable for re-resection. This recurrence (cT2N0M0) was seen at the ESD scar 57 months after a previous very low risk curative resection and was treated with radiation therapy based on the patient’s preference.

Locoregional lymph node metastasis and distant metastasis were observed in six (6/66; 9%; 95%CI 2%–16%) and four (4/66; 6%; 95%CI 0–12%) patients, respectively ( **Table 1s** ). The majority of these cases (8/10; 80%) were seen in patients with a noncurative resection, of whom five were previously additionally treated with esophagectomy (n = 4) or definitive chemoradiation therapy (n = 1) after ESD. The other two patients (2/10; 20%) developed locoregional lymph node (n = 1) or distant (n = 1) metastasis after an initially curative ESD ( **Fig. 2s** ).

### Overall and ESCC-specific survival

**Fig. 3:**
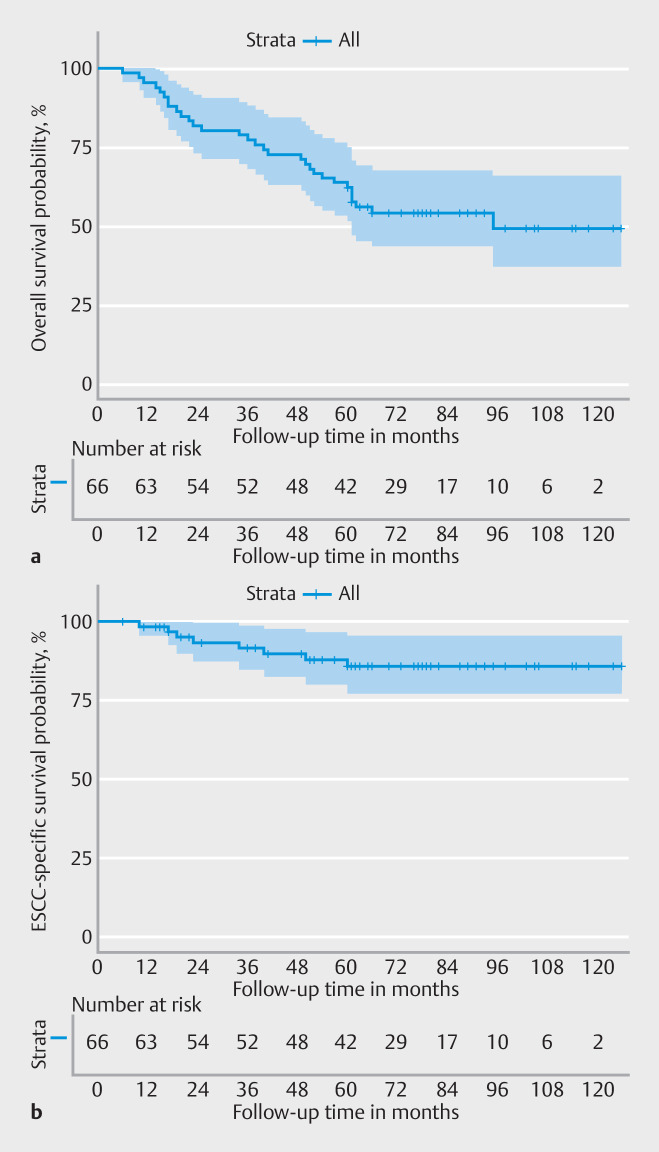
Survival probability after a technically successful endoscopic submucosal dissection (n = 66) for early esophageal squamous cell carcinoma (ESCC). **a** Overall survival. **b** ESCC-specific survival.

**Fig. 4:**
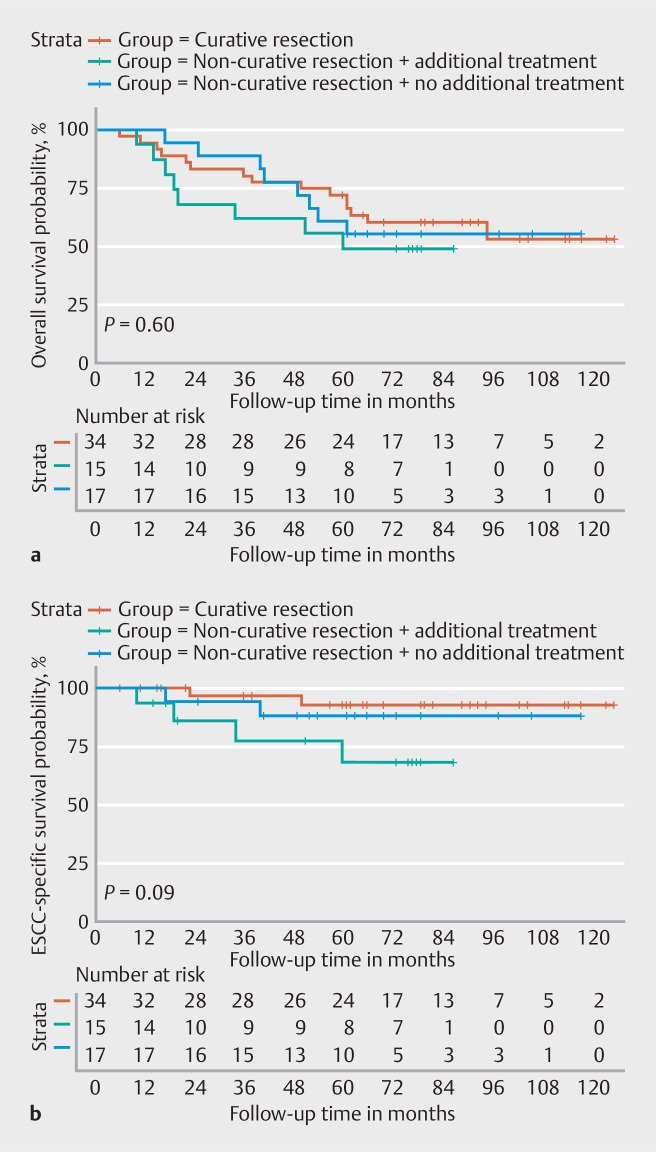
Survival probability for patients after a curative resection (n = 34), and after a noncurative resection, with (n = 15) or without (n = 17) additional treatment, for early esophageal squamous cell carcinoma (ESCC). **a** Overall survival. **b** ESCC-specific survival.

During a median follow-up duration of 66 months, 31 patients (31/66; 47%) died; 26% (8/31) were ESCC-related deaths. The 5-year overall and ESCC-specific survival probabilities were 62% (95%CI 52%–75%) and 86% (95%CI 77%–96%), respectively ( Fig. 3 ). For curative resections ( **Fig.3s** ), the difference in overall and ESCC-specific survival was not statistically significant for patients with very low risk versus low risk features ( *P* = 0.30 and *P* = 0.65, respectively). No statistically significant difference was seen in overall survival for patients with and without curative resections ( *P* = 0.60; Fig. 4 ). Although not statistically significant, ESCC-specific survival was lowest in patients with noncurative resections receiving additional surgery or definitive chemoradiation ( *P* = 0.09).

## Discussion

To the best of our knowledge, this is the largest Western cohort with the longest follow-up to date on ESD as treatment for early ESCC. In this retrospective cohort, ESD was technically feasible in 97% of patients, which is in line with previous Asian studies demonstrating similar high technical success rates (95%–100%) 12 13 14 .

One of the most striking findings was the limited long-term prognosis, with 31/66 patients (47%) dying during a median follow-up of 66 months. More importantly, 74% of these patients (23/31) died from causes other than ESCC. This high risk of competing mortality reflects the general unhealthy population affected by this disease, with tobacco and alcohol consumption being the most important risk factors 3 . Unfortunately, the retrospective nature of this study did not allow us to quantify smoking and alcohol use in our cohort.

Despite the high technical success rate, noncurative resections were frequently seen in our cohort (48%; 32/66), which is comparable to the most recent Western, single-center study consisting of 58 patients (47%) 15 . However, these rates are in contrast to Asian series, which report noncurative resection rates of less than 30% 12 16 17 18 . This difference might be explained by the fact that Asian populations undergo routine screening endoscopies of the upper gastrointestinal tract, enabling early detection of cancer. In addition, ESCC is a rare entity in Western countries, which might theoretically lead to physicians missing early lesions during diagnostic endoscopies. One may argue that our relatively high noncurative resection rate was partly influenced by patients who underwent ESD despite clinical suspicion of deep submucosal invasion, although this was only a small proportion of our cohort (4/68; 6%).

The high noncurative resection rate in our cohort emphasizes the difficulty of accurately predicting the invasion depth based on endoscopic features. Nonetheless, the general policy in the Netherlands is to consider ESD a staging procedure, as it enables accurate histopathological assessment while simultaneously being a potential, curative treatment with a minimally invasive character.

Of note, patients with ESCC typically have severe comorbidities, which may preclude a large proportion of this patient population from undergoing adjuvant surgery. This is highlighted by the fact that in our cohort only half of the patients with a noncurative resection underwent additional treatment, while the other half underwent endoscopic surveillance. In the 15 patients undergoing additional treatment after a noncurative resection, adjuvant therapy consisted of esophagectomy or definitive chemoradiation therapy in nearly all cases. Although we showed a worse ESCC-specific survival for these patients compared with patients without adjuvant therapy, this result was presumably influenced by the selection of patients with more advanced tumors for additional treatment, which is reflected by the higher percentage of ESD specimens with multiple high risk features ( **Fig. 1s** ). Nonetheless, we have shown that patients are still at risk for local recurrence and/or metastasis irrespective of additional treatment ( Fig. 2 ). In addition, there was no statistically significant difference in overall survival between this specific subgroup and patients without adjuvant treatment ( Fig. 4 ). Given this high background mortality in both groups, one may question the added value of adjuvant treatment. Consequently, the potential benefit of additional treatment should be carefully balanced against the individual risk of dying from causes other than esophageal cancer.

One of the strengths of this study is that all ESD procedures were performed by experienced endoscopists, after which all resection specimens were evaluated by expert gastrointestinal pathologists. Another strength is the use of the definitions proposed by the ESGE guideline for the short-term outcomes of ESD 6 . In addition, the meticulous data collection by dedicated research fellows minimized the risk of information bias and enabled the accurate discrimination between patients with or without additional treatment. Moreover, the occurrence of death and recurrence were checked with general practitioners or referring hospitals, resulting in high quality data for the long-term outcomes.

This study also has some limitations that need to be addressed. Patients with advanced lesions in whom esophagectomy was not possible as first-choice treatment were included in this observational cohort, which may have influenced the curative resection and recurrence rates. Regardless of our previously described efforts to collect complete data, the retrospective nature of this study holds an inevitable risk of missing data. Another limitation is the heterogeneity of staging procedures and follow-up due to a lack of a standardized protocol. As nearly half of our cohort died in the first 5 years after ESD, mainly due to causes other than ESCC, the added benefit of strict or long-term endoscopic follow-up seems questionable.

In conclusion, this study confirms the effectiveness and safety of ESD for early ESCC in a Western cohort with long-term follow-up. However, the rate of noncurative resections was substantial and overall survival of patients with early ESCC was limited, mainly due to unrelated comorbidities. Therefore, the clinical value of additional treatment after a noncurative resection should be carefully balanced for each individual patient considering that adjuvant therapy did not seem to influence overall survival in this study.

## Supplementary Material

Supplementary material Zusatzmaterial
