# Supplementary material for: Endoscopic submucosal dissection for early esophageal squamous cell carcinoma: long-term results from a Western cohort
Source: Endoscopy. 2024 Feb 7;56(5):325–33. doi: 10.1055/a-2245-7235 (PMC11582999; doi:10.1055/a-2245-7235)
Supplement: Supplementary file 1 — Supplementary material [file 10-1055-a-2245-7235_22460183.pdf_suppl.pdf.pdf]

Supplementary material

Beaufort IN, Frederiks CN, Overwater A, et al.

Endoscopic submucosal dissection for early esophageal squamous cell carcinoma: long-term results from a Western cohort.

Table 1s Characteristics of patients with locoregional or distant metastasis after endoscopic submucosal dissection.

Patient	Age	Gender	Paris classification	Tumor length, cm	Circumferential extent, %	Invasion depth	Differentiation	LVI	Radical resection	Additional treatment	Recurrence type
**Curative resection**											
1	70	Male	0-IIb	2	66	T1m3	Moderate	No	Yes	No	Locoregional lymph nodes[1]
2	70	Female	0-IIa	1	25	T1m2	Moderate	No	Yes	No	Distant metastasis[1]
**Non-curative resection without additional treatment**											
3	54	Female	0-IIa	2	100	T1sm2	Poor	No	No	No	Locoregional lymph nodes
4	72	Female	0-IIb	11	100	T1m3	Poor	No	Yes	No	Locoregional lymph nodes
5	76	Female	0-IIa	11	100	T1sm2	Good	No	No	No	Distant metastasis
**Non-curative resection with additional treatment**											
6	55	Female	0-IIb	4	55	T1sm2	Moderate	No	Yes	CRT + Surgery	Locoregional lymph nodes
7	64	Male	0-IIa	2	33	T1sm3	Poor	Yes	Yes	Surgery	Locoregional lymph nodes
8	71	Female	0-IIa	2	100	T1sm2	Moderate	No	Yes	CRT	Locoregional lymph nodes
9	65	Male	0-IIc	2	25	T1sm1	No differentiation	Yes	No	Surgery	Distant metastasis
10	66	Male	0-Ip	2	20	T1sm2	Poor	Yes	Yes	Surgery	Distant metastasis

[1]Patient developed a local recurrence or metachronous lesion with (distant) metastasis.

Abbreviations: CRT, chemoradiation therapy; LVI, lymphovascular invasion; T1m2, invasion in lamina propria; T1m3, invasion in muscularis mucosae; T1sm1, submucosal invasion <200 µm; T1sm2, submucosal invasion <500 µm; T1sm3, submucosal invasion ≥500 µm.

Supplementary material

Fig. 1s Number of histopathological high risk features (i.e. non-radical resection, poor differentiation, presence of lymphovascular invasion, and/or deep submucosal invasion) after a non-curative resection in patients who received additional treatment (n = 15) or no additional treatment (n = 17).

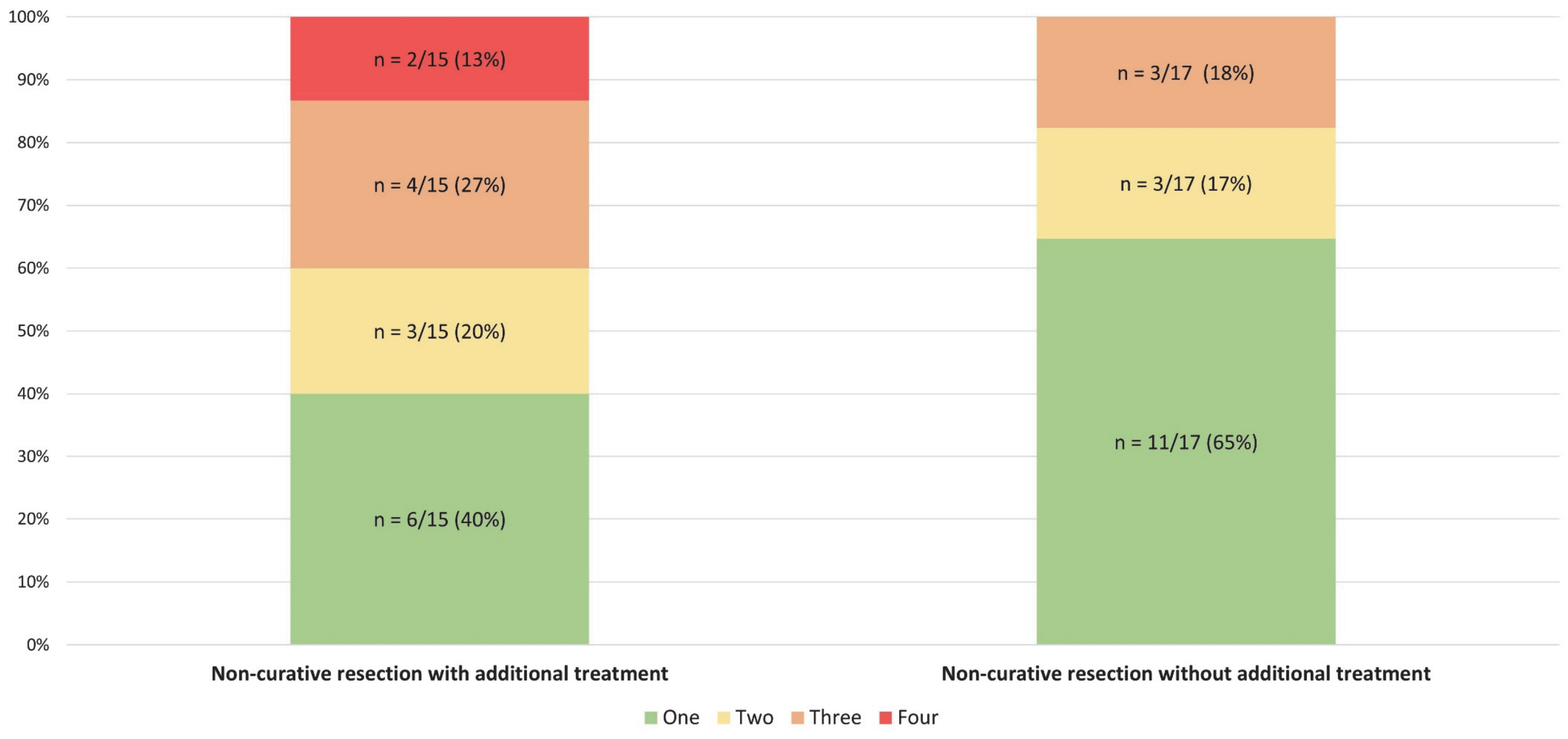

 © 2024. The author(s)

Supplementary material

Fig. 2s Local recurrence and metachronous lesion after endoscopic submucosal dissection (ESD). (A) Initially, a Paris 0-IIa lesion covering 1cm in length and 25% of circumference was detected at 30cm from the incisors. (B) After inspection with virtual chromoendoscopy, (C-D) the lesion was successfully resected through ESD, and histopathology revealed a radical resection of a moderately differentiated T1m2 ESCC without lymphovascular invasion. (E) During follow-up at three months, no dysplastic lesions were detected during careful inspection with white light endoscopy, virtual chromoendoscopy and Lugol's iodine staining. (F) Biopsies of the ESD scar however showed high-grade intraepithelial neoplasia. (G-H) Patient was rescheduled for follow-up after six months, during which a new metachronous, Paris Ip lesion was detected at 17cm from the incisors. After PET-CT showed both lymph node and distant metastases, the patient was considered inoperable and preferred to refrain from palliative chemotherapy.

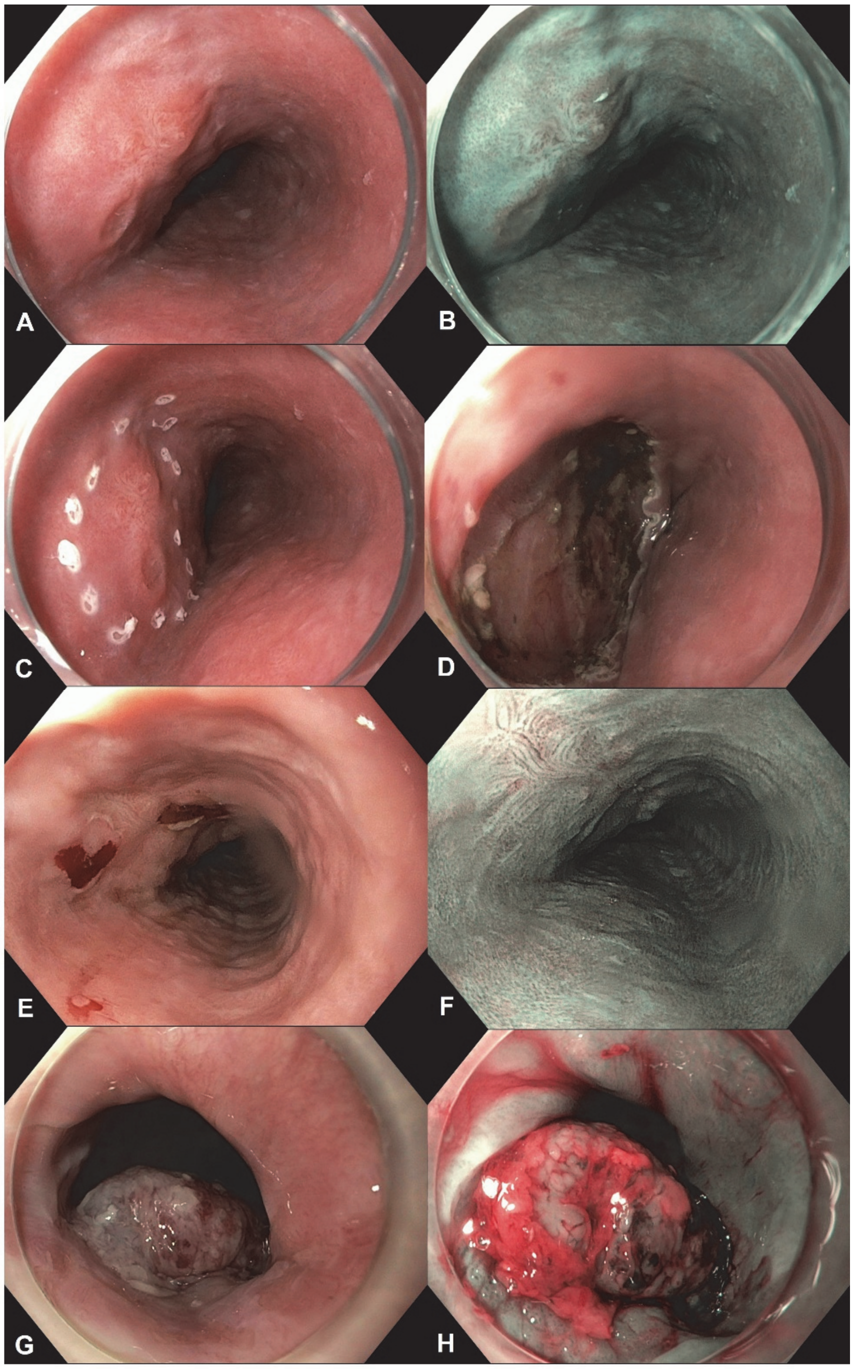

Supplementary material

Fig. 3s (A) Overall and (B) esophageal squamous cell carcinoma-specific survival probability after a very low risk curative resection (i.e. radical resection of high-grade intraepithelial neoplasia or T1m2 tumor; n = 21) or low risk curative resection (i.e. radical resection of T1m3 or T1sm1 tumor with good to moderate differentiation and without lymphovascular invasion; n = 13). None of these patients received additional surgery or chemoradiation therapy.

A

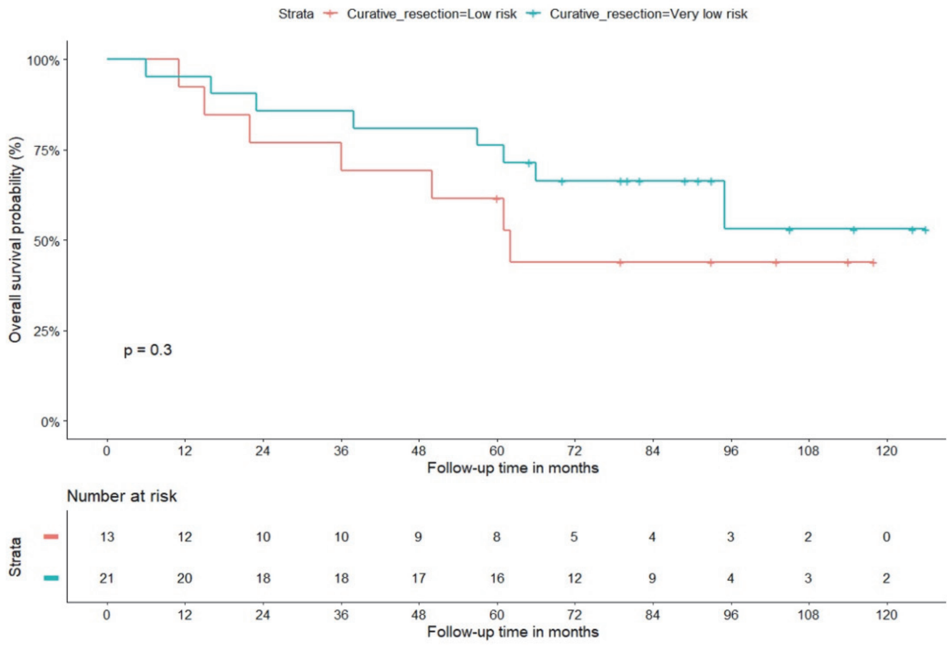

B

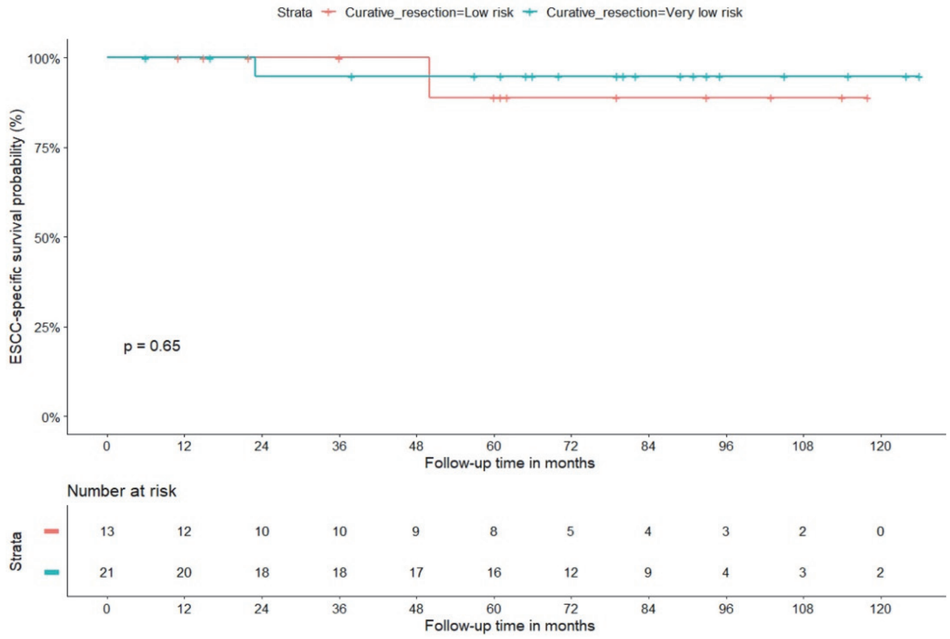

 © 2024. The author(s)
